# Five-Year Changes in Community-Level Sport Participation, and the Role of Gender Strategies

**DOI:** 10.3389/fspor.2021.710666

**Published:** 2021-10-08

**Authors:** Rochelle Eime, Melanie Charity, Jack Harvey, Hans Westerbeek

**Affiliations:** ^1^School of Science, Psychology and Sport, Federation University, Ballarat, VIC, Australia; ^2^Institute for Health and Sport, Victoria University, Melbourne, VIC, Australia

**Keywords:** sport, policy, community, sports club, women

## Abstract

**Background:** The rate of participation in community-based sport by boys and men has been double that of girls and women. Contributing to this is the fact that some sports have been traditionally male-only or at least very male-dominated.

**Aim:** The aim of this study was to investigate changes in participation in sport by sex and age across 10 major sports in Australia over a 5-year period. In conjunction with the analysis of participation trends, the gender strategies that were developed and implemented during this time are reviewed.

**Methods:** This study encompassed all sport participants registered with one of 10 State Sporting Associations in Victoria, Australia in 2015 and in 2019. Participation rates by region, age and sex were calculated. State sport and health policies relating to female participation in sport were reviewed.

**Results:** There were 749,037 registrations in 2015 and 868,266 in 2019. A comparison between 2015 and 2019 shows increases in participation for women and girls across all age groups (4–84 years), and highest increases for those aged 4 (6.6%) and 5–9 (4.7%). For boys there was a considerable decrease in participation for those aged 5–9 years (−3.8%).

**Discussion:** This study provides evidence that whilst participation in sport is still dominated by males, the gap might be gradually closing and this is in line with recent strategies and investments into sport and wider cultural developments in society. The implications of these findings are discussed.

## Introduction

### Gender^1^ Inequality and Hegemonic Masculinity in Sport

Women and girls^1^ have traditionally been, and continue to be underrepresented as both sport participants (Borgers et al., 2018; Strandbu et al., 2019; Shull et al., 2020) and in non-playing roles such as coaching, officiating, senior administrators and board members (Burton, 2015). As a matter of fact, women are underrepresented in leadership positions at all levels of sport (Burton, 2015). An international study across 45 countries reports that for sports organisations, women make up only 20% of board directors, 11% of board chairs and 16% of chief executives (Adriaanse, 2016).

Gender is arguably a highly visible position of inequality in sport (Spaaij et al., 2015). This is largely due to sport being organised around the discourses of hegemonic masculinity (Spaaij et al., 2015). Hegemonic masculinity concerns the ways that ideas about gender are embedded within social practises and how those ideals facilitate institutional power, including organising and playing sport (English, 2017). Hegemonic masculinity describes practises that legitimise men's dominant position in society, and this has traditionally played a central role in sport (English, 2017). This is partly due to the excessive focus on winning in sport (English, 2017). The effects of hegemonic masculinity are that they can harm and marginalise women in sport, and in society, in addition to harming men who do not fit the (masculine) mould (Spaaij et al., 2015; English, 2017). In addition to marginalising women, hegemonic masculinity underpins many ideals of sport including aggression, violence and high focus on competition, and fosters myths such as being tough, being competitive, winning against all odds, making sacrifices to play, and winner takes all (English, 2017). Simply stated, women participate less, and their sports are less valued in all regards (Spaaij et al., 2015). This then provides the main premise as to why we track some of the participation trends in this paper and how these may relate to new strategies to increase participation by women and girls. These strategies are aligned to increasing women and girls' participation in sport from both a participation perspective and a leadership perspective. In this paper we aim to track changes in participation in community sport by gender and age over a 5 year period and in doing so review the gender strategies that have occurred during that time within the Australian context.

### The Community Sport Landscape—Participation Trends for Women and Girls and for Men and Boys

In Australia, men and boys participate in organised community-based sport at nearly twice the rate of women and girls (Eime et al., 2020a). Analysis of participation in 10 major sports [Australian football, basketball, cricket, football (soccer), gymnastics, hockey, netball, sailing, swimming, and tennis] with a combined number of 844,992 participants aged 4–100 years showed that the participation rate for women and girls was 10% compared to 17% for men and boys. Further, breakdowns by age groupings also demonstrate this gender disparity with 64% of boys aged 5–9 years compared to 45% of girls, and 74% of boys aged 10–14 years compared to 55% of girls, playing sport (Eime et al., 2020a). An eight-year longitudinal study reported higher participation in club-based sport for young boys aged 8 at baseline (79%, compared to 65% for girls), and for adolescent boys aged 16 (71%, compared to 59% for girls) (Telford et al., 2015). A study of youth sport in Oslo (Norway) also reported that participation in sports clubs was significantly lower for girls compared to boys aged 16–18 years (Strandbu et al., 2019). In this study, the participants were classified as minority or majority, a status that was derived from where their parents were born. Those whose parents were born overseas were classified as minority status. From this study, boys were significantly more likely (40% majority and 38% minority status) to play sport than girls (25% majority and 12% minority status) (Strandbu et al., 2019).

Nevertheless, there is evidence globally that new opportunities are emerging for more women and girls to play sport. There are many reasons for this trend, including: gender equality policies and strategies (Casey et al., 2019), opportunities for women and girls to play traditionally male-only or male-dominated sports such as Australian football, football (soccer) and cricket (Ekholm et al., 2019; Ishigami, 2019; Fowlie et al., 2020), changes related to ethnicity, culture and religious beliefs (Hanlon et al., 2019; Oxford and Spaaij, 2019), and changing societal expectations and gender norms (Spaaij et al., 2015).

There has been some recent research exploring the participation of women and girls in male-dominated sports, mainly with regard to soccer internationally, and in Australian football and cricket (Ekholm et al., 2019; Ishigami, 2019; Elliott et al., 2020; Fowlie et al., 2020). A Japanese study of participation of women and girls in 15 sports and physical activities reported an overall decline in participation of 1% from 2003/2011 to 2012/2015, whereas female participation in male-dominated sports like soccer actually increased slightly (Ishigami, 2019).

A recent Australian qualitative study of 45 female Australian football community club-based players (average age of 13) and their parents investigated reasons for participating in Australian football. The authors report that many women and girls had wanted to play this sport for a long time, but previous generations of women and girls were not able to do so (Elliott et al., 2020). Further, the mothers were often supportive of their daughters' involvement because they had also desired to play during their childhood, but were not able to do so (Elliott et al., 2020). Another main driver was “thrill seeking,” and that girls were able to play a sport in which they were allowed or even expected to be aggressive, which they could not in other sports. Australian football provided an opportunity to play a heavy-impact sport. To play male-dominated sport also offered scope to develop a different personal identity, further facilitated by identification with (in this case) Australian football through media, stories and role models (Elliott et al., 2020).

Another Australian study of the barriers for women and girls to participation in cricket, a male-dominated sport, reported that the girls often lacked confidence in skills and had a desire to play with women and girls only, and that there was a need for a player pathway and specific coaching for women and girls (Fowlie et al., 2020). Some study participants reported that some of the boys did not let them play simply because they were girls. Another barrier to their involvement in cricket was a lack of visibility of female cricketers. Interviewees reported that they do not see female cricketers within their community, or female elite players, portrayed in the media. The authors argued that there is a need for female voices and for a supportive and inclusive environment and culture for all players (Fowlie et al., 2020).

Other international research highlights that women and girls often lack a presence and a voice in football (soccer) programs. In a Swedish study of sport programs and the absence of women and girls playing, the comment was made that “girls are neither heard or seen” (p1050) in football (soccer) programs (Ekholm et al., 2019). The authors concluded that the involvement of girls in these sports programs required arranging separate activities for girls, and that female coaches and role models had to be engaged. Further, the authors stated that the voices of the girls themselves need to be heard so that participation in sport can match their desires and needs (Ekholm et al., 2019).

A recent study investigating the gendered nature of sport in Colombia found that female participants were the first generation of women and girls in their community to participate in organised sport (Oxford and Spaaij, 2019). The low participation in sport for women and girls reflected social norms in Columbia, where female participation in sport is generally (still) discouraged (Oxford and Spaaij, 2019). In the two regions studied, only 20% of sport participants in Chevere and 10% in Bacano were women (Oxford and Spaaij, 2019). In these areas some girls cannot play sport due to their fathers' religious/cultural beliefs that do not allow girls to play sport as their place is to be in the house, cooking and maintaining their husband and children (Oxford and Spaaij, 2019).

A study in Sweden, investigating female participation in social inclusion football (soccer) programs, also identified that society and culture play a role, with parents not often supportive of their girls playing sport, and girls sometimes needing permission from their fathers to leave the house (Ekholm et al., 2019). The authors concluded that girls need to have female role models in sport, and “girls only” sporting activities (Ekholm et al., 2019). Another study also highlights the important role that parents can play in their children's participation and that this differs between boys and girls. Parents have been reported to place greater value, both ideologically and financially, on participation in sport for their sons compared to their daughters, and that parents were significantly more likely to positively rate the benefits of participation in sports for boys than for girls (Heinze et al., 2017). Furthermore, they investigated the parents' progressiveness of gender role beliefs. The results showed that the more progressive the parents' gender role beliefs, the more likely their daughters were to play masculine sports. The authors concluded that despite increasing opportunities for women to play sport, gender norms still pose significant barriers to equality in participation (Heinze et al., 2017).

A recent study investigating the implications of female sport policy on the community club-based sport sector reported that whilst female demand for participation in sport had increased, this had resulted in a number of challenges, including the capacity to accommodate the growth in participation in terms of availability and quality of infrastructure, and the capacity of volunteers (Casey et al., 2019). The authors also noted that retention of female participants is still a problem for community sport, and concluded that the development of gender equity in sport still requires further progress, and that a lot of community sports clubs still require assistance to create female-friendly environments to improve sport participation for women and girls (Casey et al., 2019).

### Sport Strategies That Focus on Facilitating Gender Equality

Specific sport strategies often focus on increasing participation in the under-represented or marginalised groups of society. Considering that half of the population, that is women and girls, have historically been marginalised in sport participation, many national governments, including those of Australia, New Zealand, and England, have started to develop strategic priorities and commitments to gender equality in sport, and specifically to increasing sport participation for women and girls (Sport England, 2017; Department of Health, 2018; Sport New Zealand, 2018). National sport policy in Australia acknowledges that women and girls are less likely than to men and boys to be active at health enhancing levels (Department of Health, 2018). As such, national, state/provincial and community sport strategies and investments have started to focus on increasing participation specifically for women and girls. An example of this is the Australian Government “Girls Make Your Move” campaign (Department of Health, 2018), which aims to inspire, energise, and empower young women to be more active regardless of ethnicity, size or ability, and which was based on the “This Girl Can” initiative of Sport England (Australian Government, D.o.H); Sport England, {undated #5556}.

In the Australian state of Victoria, the “Change our Game” campaign has been developed with the aim of levelling the playing field for women and girls in sport and active recreation (Victoria State Government, 2020). In 2015, the Victorian Government released a report from the independent “Inquiry into Women and Girls in Sport and Active Recreation.” This report highlighted the gender inequality in sport and recreation and outlined strategies to assist with change (Victoria State Government, 2020). This report included nine recommendations to increase participation and leadership in sport by women and girls. These included appointing ambassadors, a mandate on gender balance of boards, good governance principles, enhancing participation choice, creating female friendly facilities, providing education and training, and a focus on role models and women in sports media (Victoria State Government, 2020). From these recommendations a significant amount of funding ($7.2 million AUD) has been and continues to be directed towards improving participation and leadership in sport by women and girls (Victoria State Government, 2020). There are a number of initiatives as part of the Change our Game program including (Victoria State Government, 2020):

– Community activation grants, to support community sport and recreation organisations to deliver events that promote gender equality.
– Research grants, to undertake research relating to women and girls in sport and active recreation.
– Women in sports broadcasting program, which gives women who are interested in sports broadcasting the information and experience to further their skills in this area.
– Workforce development program, which funds initiatives that grow opportunities for women and girls to take up different roles, paid and volunteer, at all levels of sport and recreation.
– Women's Governance program, to provide formal training and development opportunities to women who are looking to strengthen their understanding of governance processes with a view of taking on board positions.
– Mandatory 40% quota for women on boards.
– Ambassador program, which is focused on connecting influential women with the broader sport and active recreation industry, in order to inspire and advocate gender equality.
– Champions program, which brings together influential leaders from the sport and active recreation sector to champion cultural change and generate leadership opportunities and experiences for women and girls.

The Victorian Health Promotion Foundation (VicHealth) has also had a strategic focus on increasing participation in sport, including social sport and active recreation, for women and girls (VicHealth, 2018). This includes strategic funding for sport organisations and program development as well as funding for the “This Girl Can” campaign which aims to inspire women and girls to get active, however, wherever and whenever they choose, without being judged (VicHealth). The objectives of the campaign are: to increase physical activity among Victorian women and girls, with a particular focus on those who are less active; and to support gender equality by challenging traditional gender roles and stereotypes in sport, and celebrating women and girls in this space (VicHealth and Latrove University, 2020). The campaign elements included (VicHealth and Latrove University, 2020): Advertising, Ambassadors, Media engagement, Social media, Campaign supporter program, Digital; Sport partnerships, Council partnerships, Communication to women, Podcast, and This Girl Can Week.

The 2020 This Girl Can—Victoria campaign inspired almost 320,000 women and girls to get active (VicHealth). Further, the campaign has inspired one in five women aged 18–65 across Victoria to get active (VicHealth).

In recent years, international sport policy has become more focused on increasing participation in sport for women and girls. Aligned to this have been many different strategies and investments aimed at increasing participation in community club-based sport, including both the traditional competitive model and more recently developed social forms of sport. The Victorian government “Change our Game” initiative is aimed at both increasing participation in playing sport and increasing female leadership in sport.

However, as Cunningham (2008) reported, the overall organisational culture in sport clubs and federations can, and probably will, continue to nurture the hegemonic masculinity that prevent women from advancing to leadership positions and worse, marginalises them. This is in line with Hoyden's (2010) findings that women in Norwegian sport organisations were prevented from taking on leadership positions, as these roles were stereotyped as masculine. Other authors have used a multilevel framework to deconstruct the role board members play in advancing gender equity (Sotiriadou and de Haan, 2019). They found that the importance of male equity champions is substantial, in that they not only pave the way for challenging stereotypes, but also are instrumental in introducing and implementing strategies, policies and constitutional change that facilitate and encourage women to take on leadership positions. They conclude that “Converging implicit thinking into explicit policy direction and doing has the power to shift the narrative of women's leadership in sport governance from gender equality to gender equity. The presence of governance trends like the use of a skills matrix and nominations committee, as well as efforts towards achieving ‘good governance' through gender diversity are indicative of changes that embrace ‘new' values and adapt organisational processes to a ‘new generation of thinking' enabling gender equity” (p. 16) (Sotiriadou and de Haan, 2019).

### Regional Differences

In addition to gender differences in sport participation, there are also consistent regional differences. In Australia, the rate of sport participation is much higher in regional areas than in metropolitan cities (Eime et al., 2016, 2019). This is related to a range of factors including population density, available space for large sporting infrastructure, and the availability of sports, with rural and regional communities generally being limited to traditional sports, while large cities provide a wider range of leisure-time activities (Eime et al., 2015a).

Victoria is a state in the southeast of Australia with a population of over 6.6 million spread across a land areas of 227,444 km^2^. Victoria's economy is the second-largest among Australian states and is highly diversified (Wikipedia). The aim of the study reported in this paper was to investigate the initial impact of the strategic direction and action by the Victorian government and health agency VicHealth, on changes in participation in sport by women and girls from 2015 to 2019.

## Methods

This study analyses and reports on club-based sport participation in 2015 and 2019 for 10 major Australian sports [Australian football, basketball, cricket, football (soccer), gymnastics, hockey, netball, sailing, swimming, and tennis], in the state of Victoria. Seven of the 10 sports are ranked within the top 10 participation sports in Australia for children and adults combined (Australian Sports Commission, 2016). These sports are part of a longitudinal study of sport participation which was initiated in 2011. The particular sports were chosen by the project funding bodies and the research team, and are among the most popular club-based sports in Victoria, ranging across gender and age of participants, and including both team and individual sports.

A sport participant, or player, was defined as a person registered with a sporting club or program that was associated with one of the 10 State Sporting Associations. In the context of community sport in Australia, the vast majority of those registered are active participants (players), and therefore these registrations provide an excellent proxy for active sport participation. This methodology has been used in a number of studies, including (Eime et al., 2016, 2019) and including longitudinal studies (Eime et al., 2015b, 2018, 2020b; Casey et al., 2019).

Data for the 10 sports were amalgamated and analysed together to create broad participation profiles and patterns whilst maintaining the confidentiality of the participant data for individual sports. Because the individual data were de-identified, individuals could not be linked from one sport to another. Consequently, an individual participant who played more than one sport was counted separately in each sport. Therefore, the counts of participants are somewhat inflated, but assuming the patterns of multiple participation are similar in the two years, this does not affect the year-to-year comparisons. The reported participation rates are strictly “participant registrations per 100 persons,” but for simplicity are referred to throughout this paper as percentages of the relevant population cohort.

Age-specific participation rates were calculated and defined as the number of player registrations in each standard 5-year age range, expressed as a percentage of the estimated resident population in that age range and defined by the Australian Bureau of Statistics (Australian Bureau of Statistics, 2017a). Separate age-specific participation profiles were calculated for each sex.

For the purpose of regional breakdowns, the research team in conjunction with research partners—State Government agency Sport and Recreation Victoria, and Victorian Health Promotion Foundation (VicHealth)—defined four regions. Each region consists of a group of local government areas (LGAs). There were two driving principles behind the designation of four regions. Firstly, the patterns of sport participation in metropolitan and non-metropolitan areas are known to differ substantially (Eime et al., 2016). Secondly, within both metropolitan and non-metropolitan areas, the projected growth in the population is very uneven (Australian Bureau of Statistics, 2017b). The Metropolitan - Growth region consists of the seven LGAs containing the four growth corridors designated by the Metropolitan Planning Authority. Six of the seven are within the current Melbourne Metropolitan Area designated by the State Government. The seventh, Mitchell Shire, is currently designated Non-metropolitan.

The Regional—Growth region consists of the LGAs containing the three largest regional centres, Geelong, Ballarat, and Bendigo, together with four LGAs which are expected, according to State Government population projections, to experience high population growth during the period up to 2021. Each of these four LGAs is on the outer periphery of one or more of Melbourne, Geelong and Ballarat.

The Metropolitan—Other region consists of the remaining 25 LGAs within the designated Melbourne Metropolitan Area.

The Regional—Other region consists of the remaining 40 LGAs outside the designated Melbourne Metropolitan Area.

In order to provide consistency across all breakdowns by region, sex, and age, those for whom residential postcode, sex or birthdate was missing or invalid (7.5% of 2019 registrations) were excluded from the analysis, and adjustments to counts were made in postcodes that were partly allocated to a Local Government Authority (LGA) outside Victoria. Age profiles of overall participation and profiles by sex and region were calculated for 2015 and 2019 and compared.

Approval to undertake the study was provided by the Federation University, Australia Human Research Ethics Committee.

### Definition of the Four Victorian regions

**Table:** 

For the purpose of regional breakdowns for this research four regions have been defined by the research team in consultation with Sport and Recreation Victoria and VicHealth. Each region consists of a group of local government areas (LGAs), listed here in alphabetical order. B, Borough; C, City; RC, Rural; City; S, Shire. There are two driving principles behind the designation of these four regions: • The patterns of sport participation in metropolitan and non-metropolitan areas are known to differ substantially. • Within both metropolitan and non-metropolitan areas, projected growth in population is very uneven. The Metropolitan—Growth region consists of the seven LGAs containing the four growth corridors designated by the Metropolitan Planning Authority. Six of the seven are within the current Melbourne Metropolitan Area designated by the State Government. The seventh, Mitchell Shire, is currently designated Non-metropolitan. The Regional—Growth region consists of the LGAs containing the three largest regional centres, Geelong, Ballarat and Bendigo, together with four LGAs which are expected, according to State Government population projections, to experience high population growth during the period up to 2021. Each of these four LGAs is on the outer periphery of one or more of Melbourne, Geelong, and Ballarat. The Metropolitan—Other region consists of the remaining 25 LGAs within the designated Melbourne Metropolitan Area. The Regional—Other region consists of the remaining 40 LGAs outside the designated Melbourne Metropolitan Area.	**Metropolitan—Growth (7)** Cardinia (S) Casey (C) Hume (C) Melton (C) Mitchell (S) Whittlesea (C) Wyndham (C) Metropolitan—Other (25) Banyule (C) Bayside (C) Boroondara (C) Brimbank (C) Darebin (C) Frankston (C) Glen Eira (C) Greater Dandenong (C) Hobsons Bay (C) Kingston (C) Knox (C) Manningham (C) Maribyrnong (C) Maroondah (C) Melbourne (C) Monash (C) Moonee Valley (C) Moreland (C) Mornington Peninsula (S) Nillumbik (S) Port Phillip (C) Stonnington (C) Whitehorse (C) Yarra (C) Yarra Ranges (S) **Regional—Growth (7)** Ballarat (C) Bass Coast (S) Baw Baw (S) Greater Bendigo (C) Greater Geelong (C) Moorabool (S) Surf Coast (S)	**Regional—Other (40)** Alpine (S) Ararat (RC) Benalla (RC) Buloke (S) Campaspe (S) Central Goldfields (S) Colac-Otway (S) Corangamite (S) East Gippsland (S) Gannawarra (S) Glenelg (S) Golden Plains (S) Greater Shepparton (C) Hepburn (S) Hindmarsh (S) Horsham (RC) Indigo (S) Latrobe (C) Loddon (S) Macedon Ranges (S) Mansfield (S) Mildura (RC) Moira (S) Mount Alexander (S) Moyne (S) Murrindindi (S) Northern Grampians (S) Pyrenees (S) Queenscliffe (B) South Gippsland (S) Southern Grampians (S) Strathbogie (S) Swan Hill (RC) Towong (S) Wangaratta (RC) Warrnambool (C) Wellington (S) West Wimmera (S) Wodonga (C) Yarriambiack (S)

## Results

**Figure 1:**
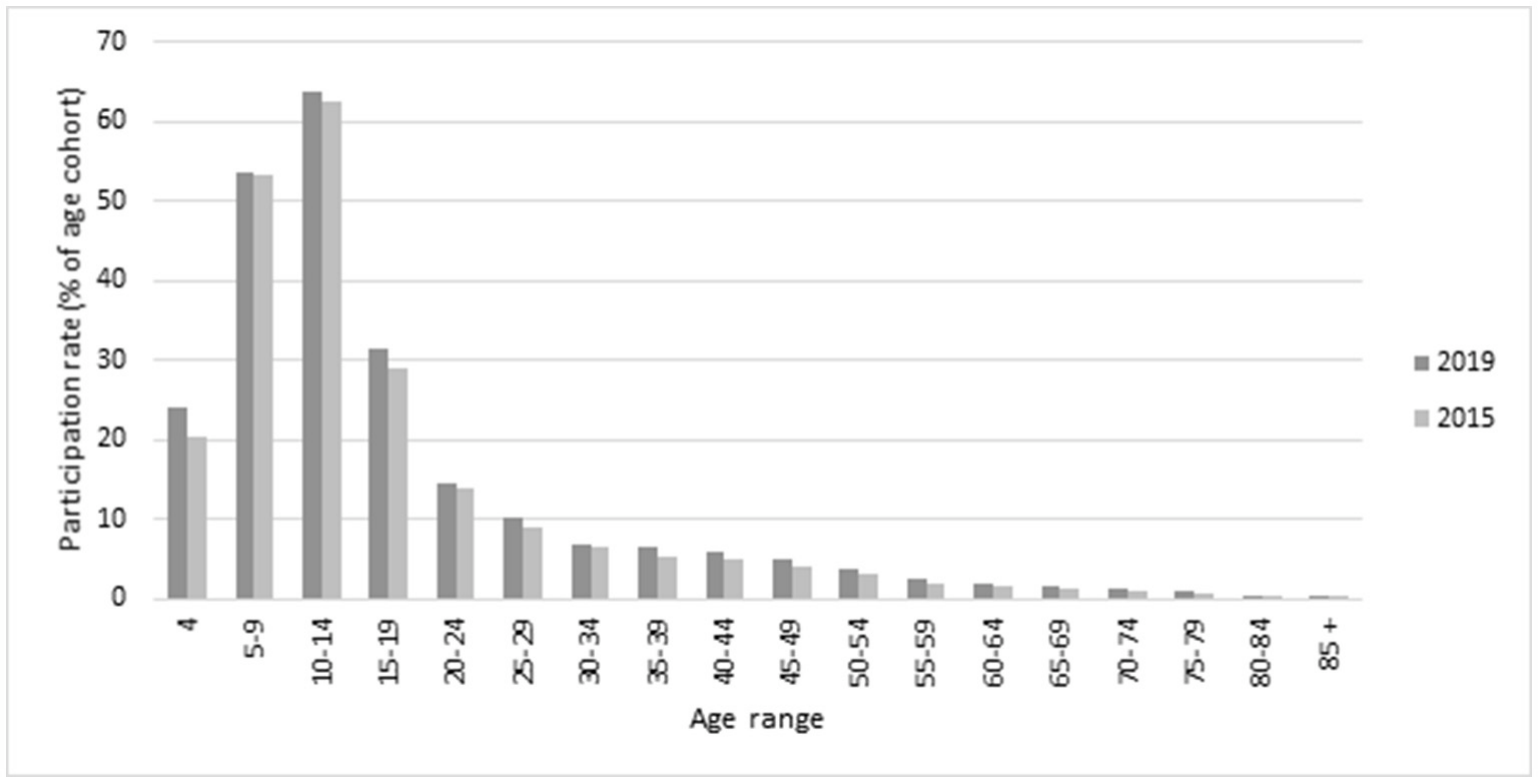
Overall participation rates in 10 sports: 2015 and 2019, Victoria: by age.

Overall participation numbers in the 10 sports increased from 749,037 in 2015 to 868,266 in 2019, representing an increase of 119,229 participants over the 5 years. This corresponded to an increase in the overall rate of participation from 12.6 to 13.4%, an increase of 0.8 of a percentage point (pp). This increase was higher for women and girls (1.1 pp) compared to men and boys (0.5 pp) (Table 1; Figure 1)^2^. Figure 1 provides an overall summary of the participation rates by age, 2015 compared to 2019.

**Table 1:** Registration counts^a^ and rates^b,c^, 2015 and 2019, Victoria: by region, sex, and age.

			**Age range**																		
**Region**	**Sex**		**4**	**5–9**	**10–14**	**15–19**	**20–24**	**25–29**	**30–34**	**35–39**	**40–44**	**45–49**	**50–54**	**55–59**	**60–64**	**65–69**	**70–74**	**75–79**	**80–84**	**85+**	**Total**
Victoria	Persons	*n* 2019	19,766	216,984	239,436	119,095	69,325	52,279	34,485	30,342	24,628	21,809	14,429	9,267	6,174	4,435	3,181	1,692	642	298	868,266
		*n* 2015	15,669	195,497	212,744	104,081	59,380	41,624	29,674	21,749	20,396	16,619	11,534	7,215	4,872	3,807	2,287	1,097	421	371	749,037
		Rate 2019 (%)	24.1	53.7	63.8	31.6	14.5	10.2	6.8	6.6	6.0	5.1	3.7	2.4	1.8	1.5	1.3	1.0	0.5	0.2	13.4
		Rate 2015 (%)	20.5	53.3	62.5	28.9	14.0	9.1	6.6	5.4	4.9	4.2	3.0	2.0	1.6	1.4	1.1	0.7	0.4	0.3	12.6
		Change 2015–2019	3.57	0.33	1.33	2.68	0.50	1.04	0.25	1.22	1.05	0.88	0.70	0.40	0.27	0.13	0.17	0.27	0.15	−0.08	0.82
	Males	*n* 2019	11,756	131,335	142,661	77,531	49,124	37,499	24,513	20,340	15,235	13,559	9,059	5,659	3,769	2,593	1,892	1,057	391	178	548,151
		*n* 2015	10,665	126,122	131,130	67,944	42,454	30,325	21,355	14,440	12,898	10,817	7,473	4,593	3,072	2,510	1,479	722	273	148	488,418
		Rate 2019 (%)	27.8	63.2	74.0	40.2	19.9	14.6	9.8	8.9	7.4	6.5	4.8	3.0	2.3	1.8	1.5	1.3	0.7	0.3	17.1
		Rate 2015 (%)	27.1	67.1	75.1	36.8	19.5	13.3	9.5	7.2	6.3	5.6	4.0	2.6	2.0	1.8	1.5	1.0	0.5	0.3	16.6
		Change 2015–2019	0.68	−3.84	−1.13	3.35	0.39	1.24	0.31	1.71	1.08	0.94	0.81	0.40	0.29	– <0.05	0.09	0.31	0.16	+ <0.05	0.51
	Females	*n* 2019	8,010	85,648	96,775	41,564	20,201	14,780	9,972	10,001	9,393	8,251	5,370	3,608	2,406	1,842	1,290	635	251	120	320,116
		*n* 2015	5,004	69,375	81,614	36,137	16,926	11,299	8,320	7,309	7,498	5,801	4,061	2,623	1,800	1,298	809	375	148	223	260,619
		Rate 2019 (%)	20.1	43.6	53.1	22.6	8.7	5.8	3.9	4.4	4.5	3.7	2.7	1.9	1.4	1.2	1.0	0.7	0.4	0.1	9.8
		Rate 2015 (%)	13.5	38.9	49.3	20.6	8.2	4.9	3.7	3.6	3.5	2.9	2.1	1.4	1.1	0.9	0.7	0.4	0.2	0.3	8.7
		Change 2015–2019	6.63	4.69	3.86	2.00	0.54	0.82	0.23	0.74	0.98	0.85	0.61	0.41	0.26	0.29	0.25	0.24	0.13	−0.14	1.13
Metropolitan	Persons	*n* 2019	3,306	36,321	37,760	19,231	12,257	8,731	6,119	5,855	3,806	2,740	1,470	842	463	303	189	98	23	12	139,525
Growth		*n* 2015	2,731	30,949	32,556	16,812	9,851	7,000	5,421	3,748	3,095	2,047	1,236	656	409	283	148	48	21	30	117,039
		Rate 2019 (%)	14.5	33.5	40.9	22.8	13.0	8.3	5.1	5.2	4.1	3.1	1.9	1.2	0.8	0.6	0.5	0.4	0.1	0.1	10.3
		Rate 2015 (%)	14.2	35.0	42.0	21.7	12.4	7.8	5.4	4.2	3.6	2.6	1.8	1.1	0.8	0.7	0.5	0.2	0.2	0.3	10.1
		Change 2015–2019	0.29	−1.46	−1.06	1.11	0.62	0.54	−0.32	0.98	0.45	0.47	0.17	0.13	– <0.05	– <0.05	– <0.05	0.17	– <0.05	−0.18	0.18
	Males	*n* 2019	1,959	22,904	24,193	13,335	9,096	6,540	4,519	4,243	2,560	1,849	994	525	288	167	103	50	13	10	93,345
		*n* 2015	1,893	20,967	21,778	11,721	7,284	5,259	4,065	2,573	2,110	1,437	823	432	252	185	96	28	11	8	80,921
		Rate 2019 (%)	16.5	41.1	51.1	30.7	18.5	12.6	7.8	7.4	5.3	4.3	2.6	1.5	1.0	0.7	0.6	0.4	0.2	0.2	13.8
		Rate 2015 (%)	19.1	46.6	55.0	29.6	17.9	11.9	8.3	5.7	5.0	3.7	2.4	1.5	1.0	0.9	0.7	0.3	0.2	0.2	14.1
		Change 2015–2019	−2.55	−5.44	−3.91	1.04	0.65	0.70	−0.50	1.68	0.37	0.52	0.28	0.08	– <0.05	−0.20	−0.12	0.14	– <0.05	+ <0.05	−0.28
	Females	*n* 2019	1,347	13,417	13,567	5,895	3,160	2,192	1,600	1,612	1,246	891	476	318	175	137	86	48	10	2	46,179
		*n* 2015	839	9,982	10,778	5,092	2,566	1,741	1,356	1,175	985	610	413	223	157	97	52	20	10	22	36,118
		Rate 2019 (%)	12.2	25.5	30.2	14.5	7.0	4.1	2.6	2.9	2.7	2.0	1.2	0.9	0.6	0.6	0.5	0.4	0.1	<0.1	6.8
		Rate 2015 (%)	9.0	23.0	28.4	13.5	6.6	3.8	2.7	2.6	2.3	1.6	1.2	0.7	0.6	0.5	0.4	0.2	0.1	0.3	6.2
		Change 2015–2019	3.27	2.51	1.81	1.01	0.39	0.33	−0.08	0.25	0.45	0.44	0.06	0.18	– <0.05	0.10	0.09	0.20	– <0.05	−0.30	0.61
Metropolitan	Persons	*n* 2019	11,664	116,147	126,350	59,388	35,516	27,482	17,628	15,151	13,363	13,102	9,308	6,145	4,110	2,978	2,222	1,220	492	216	462,481
Other		*n* 2015	8,960	106,593	116,123	52,367	30,582	21,577	14,884	10,717	10,970	9,948	7,281	4,621	3,126	2,600	1,639	822	324	223	403,357
		Rate 2019 (%)	29.2	58.4	67.4	29.6	12.0	8.6	5.9	5.8	5.8	5.4	4.3	3.0	2.3	1.9	1.6	1.2	0.7	0.3	12.9
		Rate 2015 (%)	23.2	57.5	67.2	27.5	11.5	7.5	5.5	4.6	4.6	4.5	3.4	2.4	1.8	1.7	1.4	0.9	0.5	0.3	12.1
		Change 2015–2019	6.02	0.94	0.14	2.06	0.46	1.12	0.45	1.23	1.15	0.99	0.91	0.63	0.45	0.21	0.23	0.35	0.19	– <0.05	0.87
	Males	*n* 2019	6,727	69,872	74,494	37,978	25,296	19,832	12,984	10,551	8,583	8,272	5,961	3,940	2,585	1,799	1,373	797	304	122	291,467
		*n* 2015	5,997	69,131	71,619	33,733	22,150	16,171	11,113	7,522	7,131	6,579	4,905	3,018	2,029	1,730	1,077	557	214	111	264,783
		Rate 2019 (%)	32.7	68.6	77.2	37.2	16.7	12.3	8.7	8.2	7.5	7.1	5.7	3.9	3.0	2.4	2.1	1.8	0.9	0.4	16.5
		Rate 2015 (%)	30.1	72.8	80.8	34.7	16.4	11.1	8.1	6.5	6.1	6.0	4.7	3.2	2.5	2.4	1.9	1.3	0.7	0.4	16.1
		Change 2015–2019	2.64	−4.19	−3.57	2.54	0.35	1.20	0.58	1.65	1.39	1.11	0.99	0.75	0.51	0.07	0.19	0.43	0.22	– <0.05	0.46
	Females	*n* 2019	4,938	46,275	51,856	21,410	10,220	7,650	4,645	4,600	4,780	4,829	3,348	2,205	1,525	1,179	850	424	187	94	171,014
		*n* 2015	2,963	37,462	44,504	18,634	8,432	5,407	3,771	3,195	3,839	3,369	2,377	1,603	1,098	870	562	265	110	112	138,574
		Rate 2019 (%)	25.4	47.7	56.9	21.6	7.1	4.8	3.1	3.5	4.1	3.9	3.0	2.1	1.6	1.4	1.2	0.8	0.5	0.2	9.4
		Rate 2015 (%)	15.8	41.4	52.9	20.0	6.5	3.8	2.8	2.7	3.2	3.0	2.2	1.6	1.2	1.1	0.9	0.5	0.3	0.2	8.2
		Change 2015–2019	9.63	6.31	4.01	1.65	0.56	1.04	0.34	0.81	0.91	0.92	0.85	0.50	0.40	0.35	0.27	0.27	0.17	– <0.05	1.26
Regional	Persons	*n* 2019	2,229	26,907	28,488	14,199	8,497	6,107	3,941	3,344	2,603	2,153	1,378	877	681	474	347	170	53	34	102,480
Growth		*n* 2015	1,597	20,551	21,829	11,310	6,499	4,506	3,083	2,287	2,019	1,531	1,132	752	629	434	259	122	48	59	78,646
		Rate 2019 (%)	26.9	65.6	73.3	37.2	20.3	14.9	9.8	8.8	6.9	5.3	3.6	2.2	1.8	1.3	1.1	0.8	0.4	0.2	16.3
		Rate 2015 (%)	20.6	54.9	61.8	30.3	17.3	12.9	8.7	6.5	5.3	4.1	2.9	2.0	1.8	1.3	1.1	0.7	0.4	0.4	13.6
		Change 2015–2019	6.34	10.72	11.50	6.93	2.97	2.00	1.14	2.25	1.61	1.16	0.65	0.18	+ <0.05	+ <0.05	0.09	0.15	+ <0.05	−0.21	2.69
	Males	*n* 2019	1,407	16,379	17,009	9,291	5,784	4,218	2,667	2,080	1,506	1,301	818	465	376	251	179	98	34	26	63,889
		*n* 2015	1,139	13,398	13,204	7,317	4,484	3,085	2,088	1,392	1,215	961	686	486	391	293	164	74	29	18	50,423
		Rate 2019 (%)	32.3	77.3	85.0	47.4	27.1	20.5	13.6	11.3	8.1	6.6	4.4	2.4	2.1	1.5	1.2	1.0	0.5	0.5	20.7
		Rate 2015 (%)	27.7	68.6	72.4	38.4	23.4	17.8	11.9	8.2	6.5	5.3	3.7	2.6	2.3	1.8	1.4	0.9	0.5	0.4	17.7
		Change 2015–2019	4.65	8.75	12.60	9.02	3.76	2.70	1.75	3.13	1.61	1.32	0.78	−0.24	−0.19	−0.37	−0.19	0.15	+ <0.05	0.12	3.04
	Females	*n* 2019	823	10,528	11,478	4,909	2,713	1,889	1,274	1,265	1,097	852	559	412	305	223	168	72	19	8	38,592
		*n* 2015	458	7,153	8,624	3,993	2,015	1,422	995	895	804	570	446	266	238	141	94	48	19	41	28,223
		Rate 2019 (%)	21.0	53.0	60.9	26.5	13.2	9.3	6.2	6.4	5.7	4.0	2.8	2.0	1.5	1.2	1.1	0.7	0.2	0.1	12.0
		Rate 2015 (%)	12.6	39.9	50.5	21.9	11.0	8.1	5.6	5.0	4.1	3.0	2.3	1.4	1.3	0.8	0.7	0.5	0.3	0.5	9.6
		Change 2015–2019	8.38	13.12	10.39	4.61	2.20	1.18	0.64	1.45	1.60	1.04	0.54	0.59	0.21	0.36	0.34	0.15	– <0.05	−0.39	2.40
Regional	Persons	*n* 2019	2,567	37,609	46,840	26,277	13,056	9,958	6,798	5,991	4,857	3,814	2,274	1,403	921	679	424	204	75	36	163,780
Other		*n* 2015	2,381	37,403	42,237	23,592	12,449	8,540	6,286	4,997	4,312	3,093	1,885	1,187	708	491	242	105	28	58	149,994
		Rate 2019 (%)	23.3	66.8	83.1	48.6	28.0	20.9	14.2	12.6	9.6	6.4	3.8	2.1	1.5	1.1	0.8	0.6	0.3	0.1	18.2
		Rate 2015 (%)	22.3	67.7	77.3	42.7	29.2	20.3	14.2	10.9	7.9	5.4	3.1	1.9	1.2	0.9	0.6	0.3	0.1	0.3	17.5
		Change 2015–2019	1.07	−0.84	5.87	5.94	−1.20	0.51	– <0.05	1.70	1.63	1.03	0.77	0.21	0.25	0.23	0.27	0.25	0.20	−0.11	0.72
	Males	*n* 2019	1,663	22,181	26,966	16,927	8,948	6,909	4,344	3,467	2,587	2,137	1,286	729	520	377	237	113	40	20	99,449
		*n* 2015	1,637	22,626	24,529	15,174	8,536	5,811	4,089	2,953	2,442	1,840	1,060	657	401	302	142	63	19	11	92,291
		Rate 2019 (%)	29.8	76.3	92.7	60.5	35.7	28.5	18.6	15.1	10.3	7.4	4.4	2.2	1.7	1.2	0.9	0.7	0.4	0.2	22.2
		Rate 2015 (%)	30.3	79.2	87.0	52.8	38.4	27.8	18.8	13.0	9.2	6.5	3.5	2.1	1.3	1.1	0.7	0.4	0.2	0.1	21.6
		Change 2015–2019	−0.47	−2.89	5.64	7.72	−2.65	0.62	−0.28	2.07	1.05	0.83	0.95	0.14	0.31	0.16	0.25	0.25	0.19	0.08	0.58
	Females	*n* 2019	903	15,429	19,874	9,350	4,107	3,050	2,454	2,525	2,269	1,678	987	674	401	303	187	91	35	16	64,331
		*n* 2015	744	14,777	17,708	8,418	3,913	2,729	2,197	2,044	1,870	1,252	826	531	307	189	100	43	9	48	57,704
		Rate 2019 (%)	16.7	56.7	72.9	35.9	19.1	13.0	10.0	10.3	8.8	5.6	3.3	2.0	1.3	1.0	0.8	0.5	0.3	0.1	14.3
		Rate 2015 (%)	14.1	55.4	66.9	31.7	19.2	12.9	9.7	8.9	6.7	4.3	2.7	1.7	1.1	0.7	0.5	0.3	0.1	0.3	13.4
		Change 2015–2019	2.61	1.37	6.07	4.13	−0.15	0.06	0.24	1.46	2.16	1.23	0.60	0.30	0.20	0.32	0.29	0.25	0.20	−0.23	0.85

a *Aggregated over 10 sports*.

b *Number of player registrations per 100 residents, expressed as a percentage*.

c *Rate percentages are displayed to 1 decimal place accuracy, with values >0 but <0.05 being displayed as <0.05. Changes in rates are displayed to 2 decimal place accuracy, but non-zero positive and negative differences <0.05 in magnitude are shown as + <0.05 and – <0.05, respectively*.

### Age

The participation pattern across the age profile was very similar in 2019 compared to 2015 (Figure 1; Table 1). The highest participation rate in 2019 was for those aged 10–14 (63.8%) followed by those aged 5–9 years (53.7%). Participation decreased considerably from the peak. Participation fell to 31.6% for those aged 15–19, to 14.5% for those aged 20–24 years and 10.2% for those aged 25–29 years. There were continued decreases across all age groups through to 85+.

From 2015 to 2019, the largest growth in overall participation rates was in the 4 year old age group with an increase of 3.6 pp followed by 15–19 years with an increase of 2.7 pp (Table 1).

### Sex

**Figure 2:**
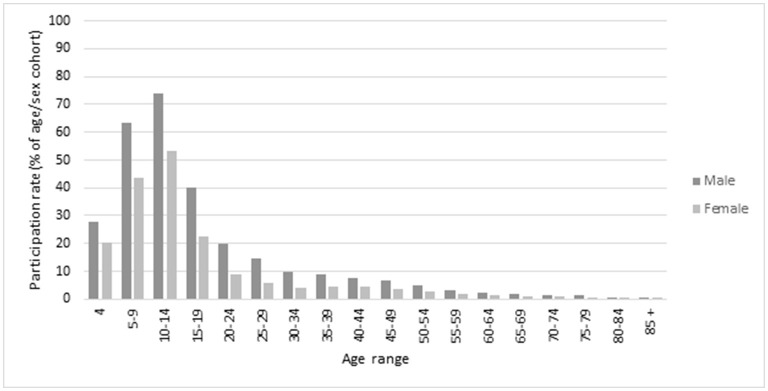
Participation rates in 10 sports, 2019, Victoria: by sex and age.

Overall, in 2019, the participation rate for men and boys was higher (17.1%) than for women and girls (9.8%). Figure 2 displays the participation rates across the lifespan by sex. Men and boys had higher participation rates than women and girls across all ages. The highest difference in the absolute participation rate was for ages 10–14 (74.0% men and boys, 53.1% women and girls). However, for several age groups within the early to middle age (20–39 years), the male participation rate was over double that of women and girls (Table 1).

The highest participation in 2019 for both males and females was in the 10–14 year age group (74.0% men and boys, 53.1% women and girls), followed by the 5–9 year age group (63.2% men and boys, 43.6% women and girls; Table 1). Overall, the male participation increased 0.5 pp whilst the female participation increased 1.1 pp.

Largest growth in the participation rate for women and girls in the 5-year period was in the 4 year age group with an increase of 6.6 pp followed by 5–9 year age group with an increase of 4.7 pp (Table 1). This was much higher than the largest growth in participation rates for men and boys, which was in the 15–19 year age group with an increase of 3.4 pp. There was a decrease of 3.8 pp for the male 5–9 year age group.

Female participation rose for all ages 4–84 2015–2019, whilst male participation decreased for ages 5–14. For ages 5–14 which represents the ages with the highest rates of participation of males in 2019 decreased between 1.3 and 3.8 pp whilst female participation increased between 4.7 and 3.9 pp for the same age range.

### Region and Gender

**Figure 3:**
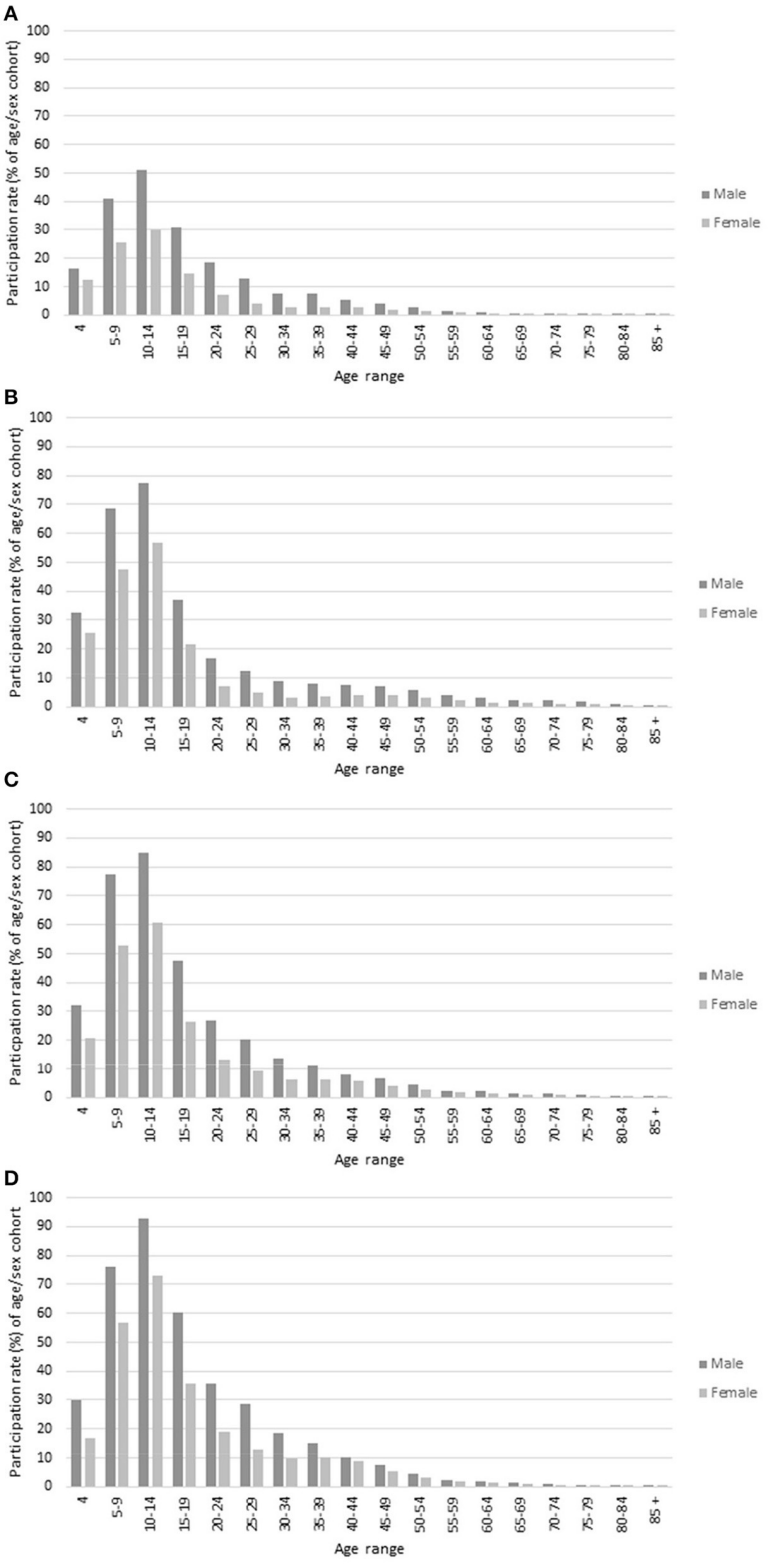
Participation rates, 2019: by region, sex, and age. **(A)** Metropolitan—Growth, **(B)** Metropolitan—Other, **(C)** Regional—Growth, and **(D)** Regional—Other.

For ages 4–49, participation rates overall were higher in regional areas than in metropolitan areas, and considerably lower in the Metropolitan—Growth area (Table 1; Figure 3). Figure 3 provides a summary of the participation rates in 2019 by region, sex, and age and for each of the four different regions (Metropolitan-Growth, Metropolitan-Other, Regional-Growth, and Regional-Other). Generally, the profiles of participation across age were quite similar across each region for both sexes. Female participation was highest for those aged 10–14 years in Regional—Other (72.9%), followed by Regional—Growth (60.9%) and Metropolitan—Other (56.9%). From these peaks, participation dropped by over 50% for the next age group 15–19 years.

The region with highest participation growth overall (2015–2019) was Regional—Growth with an overall increase of 11.5 pp (Table 1). In these areas, participation amongst men and boys increased by 3.0 pp and women and girls also increased, by 2.4 pp. Metropolitan—Other had an overall increase of 0.9 pp. In these areas, participation amongst men and boys increased by 0.5 and women and girls by 1.3 pp. Metropolitan—Growth had an overall increase of 0.2 pp. In these areas, participation amongst men and boys decreased by 0.3 and women and girls increased by 0.6 pp. Regional—Other had the largest overall increase of 0.7 pp. In these areas, participation amongst men and boys increased by 0.6 pp and women and girls increased by 0.9 pp.

### Specific Sports

Separate analyses were also conducted for each of the 10 sports. Detailed results for each sport are too extensive and repetitive to be tabulated or graphed in this paper, but the following comments provide a summary of some key comparisons.

From the 2015, data half of the sports (*n* = 5) were classified as “male-dominated” with the overall participation rate for men and boys at least double that of women and girls. Two sports were classified as “female-dominated” with the participation rate for women and girls at least double that of men and boys, and two sports gender-neutral with very similar participation rates for both sexes.

Six sports had an overall increased participation rate in 2019 compared to 2015 and four of these could be considered “male-dominated” in 2019, with at least double the participation rate for males compared to women and girls. In one sport, there was over 12 times more men and boys playing than women and girls. Three sports had the same (or <0.1 pp difference) participation rate in 2019 compared to 2015.

Seven sports had an increased participation rate for women and girls in 2019 compared to 2015. This included the four sports classified as “male-dominated,” and one sport that was “female-dominated.”

Six sports had an increased participation rate for men and boys in 2019 compared to 2015. This included one sport classified as “male-dominated.” Generally, these increases were lower than the sport specific increases for women and girls. Two of these sports reported only a very slight male increase of 0.05 pp and the highest increase was 1.0 pp.

Three of the four 2019 “male-dominated” sports reported a decrease in male participation from 2015 to 2019, and only one reported an increase, of 1.0 pp. One of the “female-dominated” sports reported a decrease in female participation from 2015 to 2019, and one reported an increase of <0.05 pp.

Given the higher participation rates for children and adolescents, the largest participation differences (growth, or decline between 2015 and 2019) were in the ages of 4–14 years. The greatest female increase in participation was within a “female-dominated” sport (increase of 4.4 pp 4 years) and a “male-dominated” sport (increase of 4.1 pp for ages 10–14 years). The greatest male increase in participation was within two “male-dominated” sports (3.7 pp for ages 10–14 and 3.4 pp for ages 15–19 years.

For ages 4–14, where the greatest sport participation occurs, and within the four “male-dominated” sports in 2019, the sport with the greatest increase in female participation across all three age groups, reported a decrease in participation for men and boys across the same age range.

## Discussion

This study provides evidence that whilst participation in sport is still dominated by males, the gap might be gradually closing and this is in line with recent strategies and investments into sport and wider cultural developments in society. Over the 5 years studied participation numbers in the 10 sports studied rose by over 119,000 which equates to an increase of 0.8 pp. When broken down by age, sex, region and specific sports there are specific nuances. Australian sport policy, as in an increasing number of nations has a focus on trying to stimulate more people to increase their levels of physical activity, and increase participation in sport, and more recently with a specific focus on women and girls (Sport England, 2017; Department of Health, 2018; Sport New Zealand, 2018). Over the 5 years analysed in this study, female participation increased at a higher rate than that of males (1.1, 0.5%, respectively). This is quite considerable considering that female participation has historically been at significantly much lower participation levels than males (Eime et al., 2016). This overarching research project measures annual change, and year-to-year differences in participation are generally quite trivial. However, this study has demonstrated that with significant gender strategies including a wide range of elements and funding from both health and government organisations that mid-term (5 year) changes can been seen. To see considerable behaviour change, it is clear that a comprehensive strategies with multiple components is required, like we have seen through the government and health initiatives in Victoria, Australia (Laverack, 2017). For behaviour change, in this case participation in sport for women and girls' strategies need to include a strong policy frameworks that creates a supportive environment and the empower of people at the community level to gain more control over making active lifestyle decisions. In this case, it would seem that the government Change our Game initiatives have provided the strong policy frameworks for example board quotas, governance programs and funding and the VicHealth This Girl Can campaign aimed at inspiring women and girls to get active are an example of effective parallel gender strategies for women and girls in sport.

There is limited comparative data reporting recent longitudinal trends in participation in community-based sport, as many studies recently published have either older data, are small sample studies, are limited to a small age range, or apply different measures of participation.

In this study the age profile was very similar in the two years studied, with highest participation for children aged 5–9 years (53, 54%) and 10–14 (63, 64%) before considerable decreases during ages 15–19 years (29, 32%). The rate increased for females predominantly for those aged 4 (increase of 7%) and 5–9 (increase of 5%). Whereas, the participation for boys decreased for those aged 5–9 years (decrease of 4%) and 10–14 years (decrease of 1%). It is promising that it is the early-sport adopters ages which have the opportunity to develop physical literacy for an active life (Westerbeek and Eime, 2021). Higher participation for children and young adolescents is consistent with other international studies (Woods et al., 2018; Kokko et al., 2019; Shull et al., 2020). Sport remains a popular leisure-time activity for children and adolescents, however with age, a decreasing number of adults are actively engaged, and this does not seem to have changed considerably over the past decade. The age profiles of sport participants has remained consistent with significant drop off during adolescence and even less adults participating (Eime et al., 2019).

The largest growth in participation was for the 4-year-olds followed by 15–19 years. This is a positive finding given that late adolescence is typically when the biggest drop in participation occurs (Eime et al., 2019; Shull et al., 2020). Previous research has consistently demonstrated the significant drop-off in participation specifically for adolescent girls, and as a result VicHealth initiated strategies that specifically targets sports to focus on participation of women and girls and specifically adolescents, aged 12–17 years (VicHealth, 2018). However, there may be issues with the large number of very young participants (preschool age) recruited into organised sport to increase sport numbers (Eime et al., 2020b). This previous recent research demonstrates very high drop-out for young children aged 4–5 years and cites supporting literature that sports should not market heavily to preschool children, as they may not be developmentally ready to participate in organised sport (Eime et al., 2020b). It has to be noted that increases in older adolescent age groups may be the result of retaining people rather than bringing new participants to sport. Stated differently, it may be that similar numbers of new participants come to participate in the sport, but that more existing participants are retained. This may be linked to VicHealth strategies which focuses on retention of participants and specifically for adolescents (VicHealth, 2018).

Whilst there were steeper increases in female participation compared to male participation, in 2019 participation for men and boys (16.6%) still remained considerably higher than for women and girls (9.8%). While a major contributing factor to this difference is the predominance in the 10 sports studied of traditionally male-dominated sports, that fact in itself is an indication of the greater participation opportunities for men and boys. The gendered results in this current study are not unexpected, in light of women and girls only recently having been provided with widespread opportunities to play many of these sports. These results are consistent with the state government and health agency gender strategies as highlighted above, and are also consistent many studies worldwide that show male participation in sport is generally higher than for women and girls (Strandbu et al., 2019; Shull et al., 2020). Even in a Nordic society such as Norway, that prides itself in having led the world in advancing gender equality, where football is the most popular sport amongst girls, participation rates of women and girls remain significantly lower than those of men and boys (Strandbu et al., 2019). The participation gap between men/boys and women/girls in less gender-equal societies remains massive, and is likely to be culturally anchored (Spaaij et al., 2015; Heinze et al., 2017; Ekholm et al., 2019; Oxford and Spaaij, 2019; Strandbu et al., 2019).

We therefore do not expect to see significant yearly trends in participation to change, only subtle and incremental shifts over time. However, in line with significant funding and strategically focusing on increasing female participation at all levels of sport (Sotiriadou and de Haan, 2019) there is evidence of a move towards more gender balance in participation. Female participation amongst 4-year-olds rose by 6.6 pp followed by 5–9 years 4.7 pp and 15–19 years 3.9 pp, which was much higher than the largest growth of boys (ages 15–19 years, 3.4%). Further, female participation increased for all ages 4–84 years, while male participation decreased for ages 5–14 years. Beyond this research there are no recent comparative studies internationally to indicate whether the gap is closing in other countries too. However, there are studies highlighting the fact that there are many cultural and societal barriers that still hinder female participation in sport, and specifically for sports that were traditionally male only/dominated (Ekholm et al., 2019; Oxford and Spaaij, 2019; Elliott et al., 2020; Fowlie et al., 2020). Several studies have highlighted that for young girls, this is the first generation able to play many (male) sports (Oxford and Spaaij, 2019; Elliott et al., 2020). In addition to the opportunity to play sport, there are many studies indicating that lack of parental support is a common barrier to participation for women and girls (Ekholm et al., 2019; Oxford and Spaaij, 2019; Elliott et al., 2020). Specific strategies are required to improve the female player pathways and coaching for women and girls (Fowlie et al., 2020), to provide role models, and to enable women and girls to have a voice in decision making about creating specific playing opportunities for them (Ekholm et al., 2019; Sotiriadou and de Haan, 2019; Fowlie et al., 2020).

In general, there were similar age profiles in each of the four regions for both genders. Participation was much higher in regional areas, and more growth was observed in Regional-Growth areas, and with larger growth for women and girls than men and boys. Participation in community club-based sport is consistently reported as being higher in non-metropolitan regions compared to metropolitan regions (Eime et al., 2016; Hoekman et al., 2017), and during adolescence those living in non-metropolitan regions are more likely to remain active through sport (Eime et al., 2019). This is related to the important social role that community club-based sport plays in regional and rural areas, and the fact that there is greater choice for a variety of leisure-time activities in metropolitan cities (Eime et al., 2015a). We deliberately defined metropolitan and regional areas of the state as “other” and “growth” to highlight the changing nature of population density, which is important to consider given the large spaces and significant planning and investment required for new sport participation infrastructure. Given that there is a continuing move of population from rural areas to the cities it is not surprising that participation decreased slightly. However, a concern is the decreased participation in metropolitan growth areas, as this is where most population growth is occurring. It is important that infrastructure and community capacity is enhanced in these population growth areas (Eime et al., 2017). The ability to increase female participation in community sport is also hindered by a lack of volunteer and infrastructure capacity (Casey et al., 2019).

The changes in participation over the five years were not consistent across specific sports. Whilst female participation increased in seven sports, and male participation in six, the female increases were largely within the male-dominated sports, and the female increases were generally higher than those of the males. Further, four of the five male-dominated sports had a decrease in male participation. If an increase in female participation is the result of special and focused strategic action and funding attention by the government, then a slight decrease in male participation may well be partly influenced by the fact that men and boys have not received this attention through strategic direction and action and broader media communication. Further, any growth in participation overall will be limited by available volunteer and infrastructure capacity.

This study had a number of limitations. Geographically, it was confined to a single state of Australia. Its scope was limited to 10 sporting codes, although these are among the most popular sports in Australia. Comparison of sports was constrained by anonymity conditions imposed by the data custodians—the state sporting organisations. While the data were comprehensive in scale—from two censuses rather than samples from two surveys—the trade-off was a limitation of detail to the basic demographic characteristics of a registration database—age, gender, and residential location, with registration providing a discrete binary indicator of participation, but with no capacity to weight participation by quantitative measures such as frequency and duration.

## Conclusion

The number of women and girls playing sport is gradually increasing for the better. An important observation from our findings is that women and girls in particular are starting to play male-dominated sports. This suggests that barriers to their previous participation may largely have been related to hegemonic masculine club cultures within these sports—that these sports “weren't for girls to play.” Such attitudes (largely by men) and perceptions (by all genders) are starting to change and these changes are in line with significant sport policy strategies and investments focused on increasing participation for women and girls, including offering a wider range of participation opportunities and options, building female friendly infrastructure, and facilitating female sport leadership. Female participation in sport internationally and in Australia is gradually increasing and developing, however there is still a long way to go. Increased opportunities will continue to create their own challenges relating to capacity of sports clubs to accommodate growth (Casey et al., 2019). Beyond that, the world has to cope with the impact of a global pandemic on participation in sport in general and on increasing the participation opportunities for women and girls in particular. Equal participation opportunities and equity in regard to leading in and benefiting from sport require multifaceted developments from both top-down and bottom-up approaches. Recommendations include ongoing strategic developments and investment, increased media attention for and exposure of female sport, social and cultural change in and around club environments, inclusive and welcoming clubs, leadership opportunities, government and community support. Furthermore, it is and important at all levels, that there is a presence and a voice for women and girls in sport. It is recommended that this longitudinal research continue in order to measure the impact of further strategic and investment initiatives across the sport sector, and to quantify the impact of COVID-19 on the sport sector, and the recovery of sport following the pandemic. Further, it is recommended that future research directly measures the impact of significant long-term sport strategies and policies on participation trends.

## Data Availability Statement

The data analysed in this study is subject to the following licences/restrictions: We are not permitted to share the data under the terms of access to the data stipulated by the primary data custodians, the Victorian State Sporting Associations.

## Ethics Statement

The studies involving human participants were reviewed and approved by Approval to undertake the study was provided by the Federation University, Australia Human Research Ethics Committee. Written informed consent for participation was not provided by the participants' legal guardians/next of kin because: this project utilised de-identifiable data and was secondary data analysis.

## Author Contributions

The data used in this study stems from the Sport Participation Research Project which is conducted by the team comprising of RE, MC, JH, and HW. RE and HW conceptualised the paper, and developed the manuscript. MC conducted the analysis and produced the results. JH and MC have contributed to the preparation of the manuscript. All authors have read the final version.

## Funding

This research stems from the Sport Participation Research Project which is funded by VicHealth and Sport and Recreation Victoria.

## Conflict of Interest

The authors declare that the research was conducted in the absence of any commercial or financial relationships that could be construed as a potential conflict of interest.

## Publisher's Note

All claims expressed in this article are solely those of the authors and do not necessarily represent those of their affiliated organizations, or those of the publisher, the editors and the reviewers. Any product that may be evaluated in this article, or claim that may be made by its manufacturer, is not guaranteed or endorsed by the publisher.

## Footnotes

^1^Social policies regarding sexuality are generally framed in terms of “gender,” i.e., sexual identification or masculinity/femininity, but data collection by sporting organisations and statistical agencies alike is generally framed in terms of “sex,” i.e., biological characteristics. In this paper both terms are used as and where appropriate. Further, the categories “male” and “female” apply to both gender and sex. However, in accordance with the current practice of public agencies in Victoria, Australia, in this paper the terms “men and boys” and “women and girls” are used rather than the plural noun forms “males” and “females”.

^2^Because the data analysed in this study are derived from two “censuses” of all registrations in the 10 sports, the participation rates cited throughout this paper are not sample estimates, but (apart from imperfections in the source databases) true population quantities—parameters of the various segments of the Victorian population. As such, statistical inference procedures (significance tests, confidence intervals) are neither necessary nor applicable.
