# Corrigendum: Five-Year Changes in Community-Level Sport Participation, and the Role of Gender Strategies

**DOI:** 10.3389/fspor.2021.798271

**Published:** 2021-11-16

**Authors:** Rochelle Eime, Melanie Charity, Jack Harvey, Hans Westerbeek

**Affiliations:** ^1^School of Science, Psychology and Sport, Federation University, Ballarat, VIC, Australia; ^2^Institute for Health and Sport, Victoria University, Melbourne, VIC, Australia

**Keywords:** sport, policy, community, sports club, women

In the original article, there was a mistake in Figure 3. The wrong graphs were used and they were in the wrong order. The corrected Figure 3 appears below.

**Figure 3:**
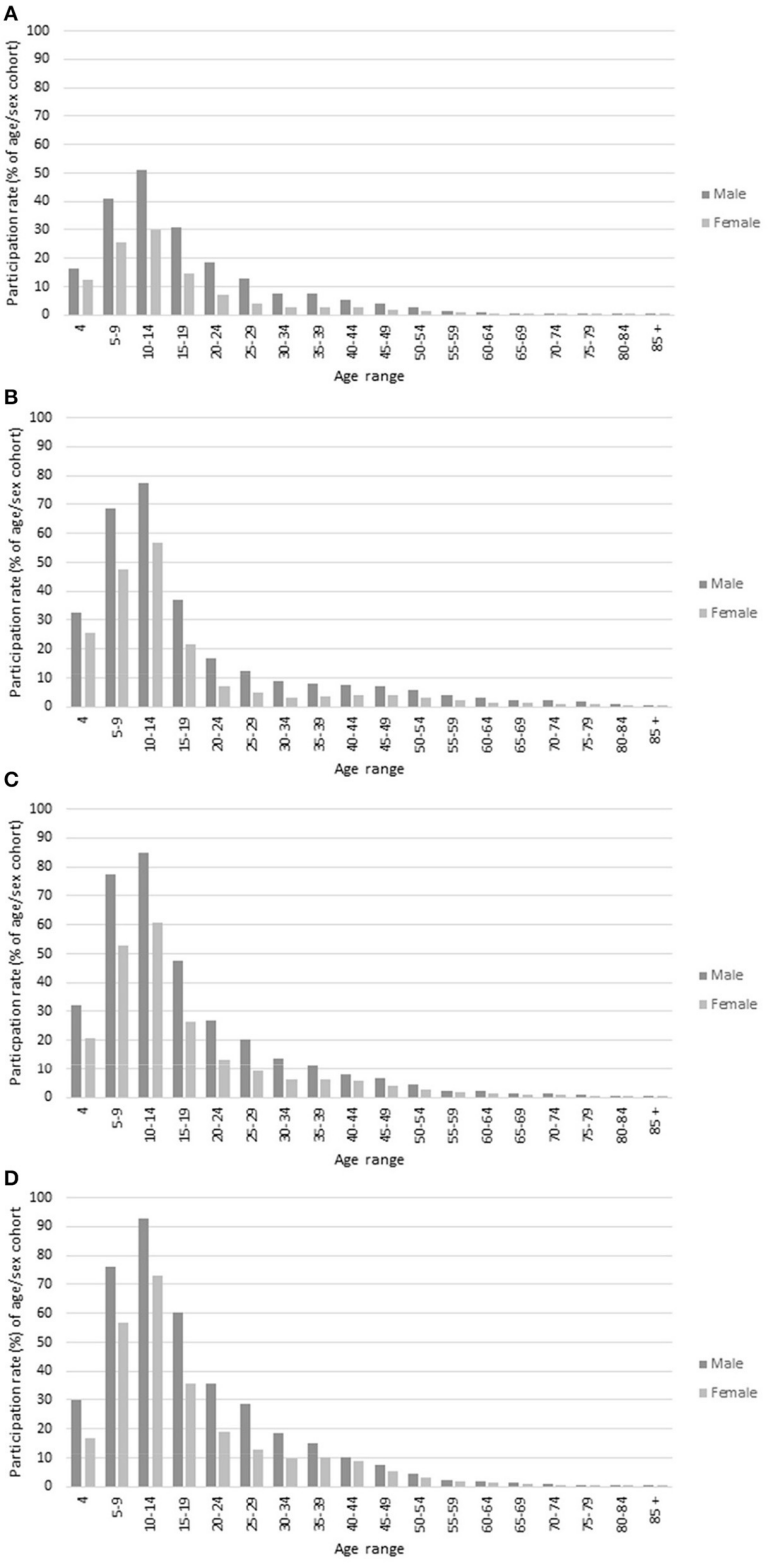
Participation rates, 2019: by region, sex, and age. **(A)** Metropolitan—Growth, **(B)** Metropolitan—Other, **(C)** Regional—Growth, and **(D)** Regional—Other.

In addition, there was an error in the Abstract, Aim paragraph, where it states it was a 4-year study period. After revisions and inclusion of an additional year of data this was changed to a 5-year study, as per the title. The corrected paragraph appears below.

“The aim of this study was to investigate changes in participation in sport by sex and age across 10 major sports in Australia over a 5-year period. In conjunction with the analysis of participation trends, the gender strategies that were developed and implemented during this time are reviewed.”

The authors apologize for these errors and state that this does not change the scientific conclusions of the article in any way. The original article has been updated.

## Publisher's Note

All claims expressed in this article are solely those of the authors and do not necessarily represent those of their affiliated organizations, or those of the publisher, the editors and the reviewers. Any product that may be evaluated in this article, or claim that may be made by its manufacturer, is not guaranteed or endorsed by the publisher.

